# Association of Systemic and Ocular Comorbidities with Visual Recovery in Patients with Postoperative Corneal Oedema: A Real-World Study of Cataract Surgery Outcomes

**DOI:** 10.3390/jcm15156100

**Published:** 2026-08-05

**Authors:** Mirna Džaja Lozo, Miro Vuković, Petra Rizvan, Ana Marušić, Ljubo Znaor

**Affiliations:** 1 Department of Research in Biomedicine and Health, Centre for Evidence-Based Medicine, University of Split School of Medicine, Šoltanska 2A, 21000 Split, Croatia; mirna.dzaja@gmail.com (M.D.L.); miro.vukovic@mefst.hr (M.V.); petra.rizvan@gmail.com (P.R.); 2 Science Department, University Hospital of Split, Spinčićeva 1, 21000 Split, Croatia; ljznaor@kbsplit.hr; 3 Division of Posterior Eye Segment, Department of Ophthalmology, University Hospital of Split, Spinčićeva 1, 21000 Split, Croatia

**Keywords:** cataract surgery, phacoemulsification, corneal oedema, visual recovery, ocular comorbidities, systemic comorbidities, real-world evidence

## Abstract

**Background/Objectives:** To evaluate the combined effect of systemic and ocular comorbidities on postoperative recovery after phacoemulsification by assessing corneal status, visual acuity outcomes, and visual recovery dynamics using a real-world data study approach. **Methods:** This retrospective analysis was conducted on patients undergoing small-incision phacoemulsification. Patients were categorised based on the presence of ocular and systemic comorbidities into four groups: those with only ocular diseases, those with only systemic diseases, those with neither ocular nor systemic diseases, and those with both ocular and systemic comorbidities. For evaluation of postoperative recovery and the impact of comorbidities, we analysed postoperative visual acuity and the recovery of visual acuity after the surgery. **Results:** Our results indicate that ocular comorbidities are associated with slower early postoperative visual recovery, while systemic comorbidities alone did not have a significant impact on visual acuity values and recovery. However, the differences between comorbidity groups decreased over time following surgery and postoperative visual acuity outcomes became comparable across groups. This suggests that regardless of the initial disease burden, the long-term effectiveness of cataract surgery has benefit and leads to improvement in visual function. **Conclusions:** Patients with ocular comorbidities, because of the slower postoperative recovery based on visual acuity, should receive closer monitoring in the early postoperative period. These findings can help in the optimisation of the postoperative follow-up protocols and reduce the healthcare burden associated with cataract management.

## 1. Introduction

Eye cataracts not only affect the quality of life but also have an impact on long-term healthcare costs [1]. The number of people affected by cataracts is expected to grow significantly as the global population ages and life expectancy continues to rise [2]. It has been reported that over 70% of people aged 80 and older had clinically significant cataracts [3]. Surgery is still the only effective treatment, and phacoemulsification (PHACO) is currently considered the gold standard therapy [4,5,6]. Although surgical techniques have significantly improved over the years, reducing the incidence of intraoperative and postoperative complications [7], postoperative follow-up is a common practice. Recent studies show that postoperative follow-up can be less intensive with a reduced number of postoperative visits for patients with no complications [8,9]. Following this approach, it is necessary to find a way to stratify patients with systemic and ocular comorbidities into those who are low risk patients from those who are possibly high-risk patients and, in that way, contribute to better rationalisation in healthcare resources. Still, some complications can occur, and the most common include transient corneal oedema [10], temporary elevations in intraocular pressure (IOP) [9], and macular oedema [11]. Transient corneal oedema is one of the most common early postoperative complications, which presents as stromal and epithelial clouding [12]. It appears as a result of stromal inflammation caused by endothelial cell dysfunction before or during the surgery [13]. This condition reduces corneal transparency, delays recovery, and may lead to slightly worse final visual outcomes [14,15]. Clinical studies suggest that the incidence of postoperative corneal oedema is likely underestimated, as mild or subclinical cases often go undetected [16].

One of the reasons for the occurrence of transient corneal oedema after cataract surgery is decreased preoperative endothelial cell count. Studies showed that endothelial cell count decreases by approximately 0.6% per year [17] and when the cell count falls below 500 cells/mm^2^ [18] or, according to some authors, even below 700 cells/mm^2^ [19], the risk of developing corneal oedema following phacoemulsification significantly increases. Factors such as age, gender [20], glaucoma [21], shallow anterior chamber with history of previous ocular procedures [13,22], trauma, and systemic conditions like diabetes contribute to endothelial cell loss [23,24]. Given the age at which cataract usually occurs, the majority of patients undergoing cataract surgery have at least one of the systemic conditions, including diabetes, risk factors for cardiovascular comorbidities, autoimmune disorders, and thyroid dysfunction. Other ocular comorbidities, including glaucoma, macular diseases, corneal disorders, and pseudoexfoliation syndrome (PEX), are also common in this patient cohort and have been shown to influence PHACO surgical outcomes [25,26,27].

Most of the studies on surgical outcomes of cataract surgery are controlled studies where analyses are conducted on a sample of patients to assess the connection with postoperative corneal oedema or poorer visual acuity outcomes. Such studies often do not include patients with eye disease [28,29] or they analyse only the patients operated on by a single experienced surgeon [30] or analyse only patients with one or two systemic comorbidities [23,29] but do not clearly define whether they had other systemic comorbidities [31]. Therefore, it is unrealistic and not reflective of real-world settings to isolate a single disease or to study only patients with one well-defined condition. In clinical practice, most patients present with more than one coexisting comorbidity, and many are unaware of the exact names or nature of their conditions. Given the importance of thorough preoperative evaluation and its potential impact on postoperative results [32,33], as well as long-term healthcare benefits, we analysed real-world data on how systemic and ocular comorbidities alone and in mutual combination influence the appearance of postoperative corneal oedema and visual acuity outcomes after cataract surgery. Such findings can potentially inform future practice in terms of patient-tailored follow-up approaches after surgery, reducing unwanted outcomes in high-risk patients and reducing healthcare costs related to follow-up visits in patients with a low risk of complications and good postoperative recovery.

## 2. Materials and Methods

### 2.1. Study Design

This was a retrospective follow-up study of patients who underwent cataract surgery at the Ophthalmology Department of the University Hospital of Split, the largest healthcare centre in Dalmatia County in Croatia. About 1 million citizens of the Republic of Croatia, and about 500,000 inhabitants of the southern part of neighbouring Bosnia and Herzegovina, gravitate towards the regional hospital, as well as around 500,000 tourists during the tourist season [34].

### 2.2. Patients

We retrospectively reviewed the electronic health records of 347 patients who underwent cataract surgery at the University Hospital of Split in November and December 2022. Postoperative clinical data generated during routine care and recorded in the electronic health records through 31 December 2023 were included. Ethics approval was obtained on 29 April 2024, before research-specific data extraction and analysis commenced. Postoperative visual status was followed by reviewing patients’ electronic health records up to the end of 2023. In each patient’s electronic health record, we extracted notes from the preoperative clinical examination, the first postoperative clinical examination with visual acuity measurement results (which usually occurred within 8–10 days after the surgery), and the final postoperative clinical examination with visual acuity measurement results. Because of the waiting times for follow-up appointments, some patients underwent their final postoperative examination toward the end of 2023. Clinical examinations that occurred between the first and final postoperative follow-up were not included in this analysis.

For analysis, the patients were grouped into the following groups:

(1) Those without systemic and/or ocular comorbidities (n = 61);
(2) Those with both systemic and ocular comorbidities (n = 59);
(3) Those with systemic but without ocular comorbidities (n = 182);
(4) Those without systemic but with ocular comorbidities (n = 13).

### 2.3. Ethics Considerations

The study was approved by the Ethics Committee of the University Hospital of Split, Croatia (Class No.: 500-03/23-01/199; Document registration No.: 2181-147/01-06/LJ.Z.-24-04; approval date: 29 April 2024). All clinical information analysed in this retrospective study had previously been generated and documented during routine patient care. Research-specific access to the medical records, data extraction, anonymization, and analysis commenced only after ethics approval had been obtained. To ensure transparent reporting, the study was presented according to the Strengthening the Reporting of Observational Studies in Epidemiology (STROBE) guidelines [35].

### 2.4. Inclusion and Exclusion Criteria

The inclusion criteria were all patients undergoing cataract surgery with the implantation of the intraocular lens (IOL) in the University Hospital of Split in November and December 2022. Out of 347 patients, the final analysis included 315 patients with a history of systemic disease (diabetes mellitus, risk factors for cardiovascular comorbidities, autoimmune comorbidities, and thyroid disorders) and/or eye comorbidities (glaucoma and PEX) and had data from at least one postoperative visual acuity. The exclusion criterion was incomplete data from the postoperative period.

### 2.5. Surgical Procedure

Due to the retrospective nature of the study, the surgical techniques used by individual surgeons were not analysed, as these typically vary based on cataract severity and personal surgical preferences. All patients underwent PHACO, performed under topical anaesthesia. The surgical approach was similar for all operators, with some variations. After topical anaesthesia and corneal incision, viscoelastic was put into the anterior chamber, followed by paracentesis. Continuous curvilinear capsulorhexis was created with a cystotome or with capsulorhexis forceps, depending on the surgeon’s preferences. After hydrodissection and hydrodelineation were performed, viscoelastic was again injected just before PHACO. Following that step was the nucleus extraction. After automated irrigation-aspiration to remove the cortex, the capsular bag was filled with viscoelastic, and the intraocular lens was inserted. In the end, the viscoelastic material was aspirated, and incisions were closed with hydration and the application of an intracameral antibiotic.

### 2.6. Medications

Standard postoperative therapy included a combination of antibiotic and corticosteroid eye drops and ointments. In selected cases, non-steroidal anti-inflammatory (NSAID) eye drops (Yellox eye drops, 0.9% bromfenac, Bausch + Lomb (Gurugram, India)) or subconjunctival dexamethasone were administered based on the physician’s assessment at individual follow-up visits and depending on the clinical condition of the eye.

### 2.7. Postoperative Follow-Up

The standard postoperative follow-up protocol is as follows: the first examination is performed in the afternoon on the day of surgery, the second on the day after surgery, and the next visit is scheduled 7–10 days postoperatively, depending on the judgement of the attending ophthalmologist. A further follow-up is typically performed three weeks after surgery.

The examination at which refraction is determined is considered the final postoperative visit. There is no fixed interval between this visit and the preceding one, as scheduling depends on appointment availability in outpatient clinics; therefore, the timing of the final postoperative examination may vary.

### 2.8. Data Collection

Data were collected from patients’ medical records and surgical protocols. All extracted data were anonymized and stored in a spreadsheet accessible only to the principal investigator. Data on systemic and ocular comorbidities were also collected, based on both documented diagnoses and the medications patients were using.

We collected the information on systemic comorbidities: diabetes mellitus (regardless of the treatment type or diabetes type), cardiovascular conditions/risk factors (arterial hypertension, hyperlipidaemia, arrhythmias, prior myocardial infarction, cerebrovascular incident, percutaneous coronary interventions, and coronary artery bypass grafting), autoimmune comorbidities (all types put in one group), and thyroid disorders (hypo- and hyperthyroidism), and for ocular comorbidities we focused on data for patients with glaucoma (all types) and PEX.

### 2.9. Postoperative Corneal Status

After the examination on the slit lamp, all patients who had a hazy or less transparent cornea or Descemet’s folds were included in the oedema group. Corneal status was categorised in two ways based on the similar categorization we found in another study [36]. The first classification was based on terms having a healthy (transparent) or pathological (if in medical records, data that the cornea was hazy, less transparent, or had Descemet folds). Second was in three groups according to clinical status: (1) transparent cornea, (2) less-transparent (hazy) cornea, (3) Descemet’s folds.

### 2.10. Visual Acuity

Visual acuity was assessed using Snellen charts, converted to LogMAR for statistical purposes, following methodologies described in previous studies [9,25,37]. For conversion, we used the method from Tiew, Lim et al. 2020 [38], considering their respective advantages and limitations. We assessed the visual acuity (VA) at different time points: preoperative (VA0) on the day before surgery; first postoperative (VA1) at one of the first postoperative control visits when the patient was able to read the Snellen chart; and the final postoperative (VA2), which represents the last available visual acuity in the accessible hospital medical records, and which differs in timing among patients, since some underwent final refraction in the hospital after cataract surgery, while others probably did not due to further follow-up in external institutions.

Visual acuity recovery was calculated as the change from preoperative visual acuity (VA0) to first postoperative visual acuity (VA1) and to final postoperative visual acuity (VA2), expressed as VA1–VA0 and VA2–VA0.

### 2.11. Statistical Analysis

The data were presented as absolute and relative frequencies along with arithmetic mean/standard deviations and median/interquartile ranges (IQR). Data distribution was tested using the Shapiro–Wilk test for Normal distribution. Differences between two nominal variables were tested using the Chi-squared test, while differences between multiple independent variables were tested using the Kruskal–Wallis test, having in mind the asymmetric data distribution. For analysing possible predictive factors of postoperative visual acuity, ANCOVA analysis was used. In all statistical tests, statistical significance was set at *p* = 0.05.

## 3. Results

Among 347 patients who underwent cataract surgery in the observed period, 315 patients with complete clinical data were included in the final analysis. The median age was 73 years (IQR 67–79), with 60% (n = 190) female patients.

The surgeries were performed by 10 different surgeons, and the median number of operations per surgeon was 21 (IQR 7–50), ranging from 1.0% to 26.7% of surgeries from the fewest to the most surgeries done per surgeon. Between the first postoperative follow-up and the final postoperative visit, some patients had additional visits, depending on the individual clinical judgement made by the attending ophthalmologist. The median number of added visits due to Descemet folds was 1 (IQR 1–3).

There was a statistically significant difference in age between the groups of patients. The oldest patients were those having both systemic and ocular comorbidities, and the patients having just systemic comorbidities without ocular comorbidities (Table 1). There was also a small but significant difference in the gender of the patients, with older women predominating in the groups with systemic and ocular comorbidities and with only systemic comorbidities (Table 1).

All patients had their first preoperative visit on the day before the surgery. The first postoperative follow-up was 8.00 days (IQR 8.00–10.75) after surgery, and the final postoperative visit was approximately 163 days (IQR 59.0–206.0) after the surgery.

There were no significant differences in preoperative endothelial cell count among the four patient groups (Table 1). The four patient groups also did not differ in the overall corneal status (healthy vs. pathological cornea) or the postoperative clinical corneal status (transparent vs. non-transparent cornea and Descemet folds) (Table 2).

Patients with only ocular comorbidities had significantly worse visual acuity at the first and second postoperative controls (Table 3).

When analysing visual acuity recovery, only the VA1–VA0 interval showed a significant difference and only for the patient with only ocular comorbidities, who had the smallest improvement in visual acuity (Table 4). There were no differences among patient groups for the VA2–VA0 interval (Table 4).

Analysis of covariance (ANCOVA), controlling for age, gender, and preoperative endothelial cell count as covariates, showed that patients’ group did not significantly predict VA1 (LOGMAR_2) (F = 2.438, df = 3, *p* = 0.065; R^2^ = 0.031), although the effect approached significance. For VA2 (LOGMAR_3), the patient group was a significant predictor (F = 2.863, df = 3, *p* = 0.038; R^2^ = 0.039). None of the covariates (age, gender, preoperative endothelial cell count) reached significance in either model. Pairwise comparisons between individual groups for VA2, however, did not reach significance after Bonferroni correction, suggesting a diffuse rather than pairwise-localized group effect.

## 4. Discussion

This study evaluated the impact of systemic and ocular comorbidities on corneal status and visual acuity outcomes following cataract surgery in a real-world clinical setting. The main finding is that while ocular comorbidities are associated with poorer early postoperative visual acuity and slower visual recovery, long-term visual outcomes were similar and comparable across patient groups, regardless of the presence of systemic and/or ocular comorbidities. These findings suggest that, although cataract surgery remains effective and beneficial, the approach should be tailored to each patient, having in mind the comorbidities that can influence the speed of recovery and rate of complications.

Regarding the influence of endothelial cells on cataract surgery outcomes, the previous literature has shown a significant influence of lower preoperative endothelial cell count [13,39], suggesting that cell count can be a potential predictor of postoperative visual acuity [15]. In our study, this problem was approached indirectly by utilising the connection between systemic and ocular comorbidities and preoperative endothelial cell count. Previous literature has already described the decreased cell count in patients with some ocular comorbidities [40,41] as well as systemic comorbidities (diabetes, cardiovascular comorbidities and thyroid disorders) [13,28,41,42]. This is in contrast to our results, where, although patients with only systemic comorbidities and patients with both systemic and ocular comorbidities had lower mean endothelial cell counts, preoperative endothelial count did not significantly differ between groups. This suggests that baseline corneal endothelial reserve was comparable across comorbidity groups and that comorbidity status alone did not appear to significantly influence preoperative endothelial cell count. In contrast to some previous reports that put preoperative endothelial cell count as the key reason for early postoperative corneal oedema [43], our study did not demonstrate significant differences in endothelial cell density between comorbidity groups, nor did endothelial cell count predict visual outcomes. This smaller impact on endothelial cells we can explain with advances in surgical technique, such as refined phacoemulsification energy management and improved viscoelastic devices, and better perioperative management [41,43,44].

These results indicate that the comorbidity burden alone does not necessarily translate into clinically relevant differences in corneal recovery. These findings suggest that the interpretation of preoperative endothelial cell count, while being an important approach to patient-tailored care, needs to be interpreted in the context of systemic and ocular comorbidity burden rather than as a standalone risk factor. This is clinically relevant because endothelial cell density, according to previous studies, is a key factor of postoperative corneal clarity and recovery [45,46] and reduced endothelial cell counts are associated with early postoperative corneal oedema and delayed visual recovery [39,47,48].

Building upon the analysis of ocular/systemic comorbidity burden, our study found no difference in corneal outcomes between patient groups with comorbidities, regardless of whether corneal transparency was analysed as the binary outcome (healthy vs. pathological corneal status) or according to the level of corneal transparency (transparent, blurred, Descemet folds). Although these findings seem contradictory to the previous findings where individual comorbidity is usually assessed as an important risk factor [23,40], a possible explanation can be found in contemporary surgical techniques, including small-incision phacoemulsification [36] and viscoelastic use, which may contribute to reduced corneal trauma even in higher-risk patients [39,49,50]. Furthermore, the current healthcare approach that includes targeted postoperative therapy has further reduced the incidence of postoperative oedema in high-risk patients, such as the use of NSAID drops (Yellox eye drops, 0.9% bromfenac, Bausch + Lomb) in patients suffering from diabetes [51,52], has further decreased the differences in outcome quality between high- and low-risk patients. Importantly, most previous studies have analysed isolated comorbidities in selected patient populations, often focusing strictly on diabetes, cardiovascular comorbidities among systemic comorbidities, or conditions like pseudoexfoliation syndrome and glaucoma among ocular comorbidities [12,23,36,47,53,54,55]. This approach has emphasised the importance of analysing the effect of each comorbidity individually and has excluded all patients who had a combination of comorbidities. Although this disease-centred approach provides insight into the detrimental effect of a single disease, it does not reflect the interplay of multiple conditions that patients usually suffer from and can overemphasise the influence of a single risk factor. This methodological gap is particularly notable with respect to thyroid and autoimmune comorbidities, where the evidence base remains sparse [29]. In contrast, our study reflects the cumulative and interacting effects of multiple conditions in routine clinical practice. By evaluating patients with complex comorbidity profiles in a real-world setting, these findings suggest that contemporary surgical and perioperative management can reduce the influence of individual risk factors on corneal recovery.

To achieve a more granular analysis of influencing factors, we took visual acuity recovery as an important component of this study. We compared the preoperative acuity levels with first and final postoperative levels of visual acuity by subtracting the two values to obtain a more dynamic assessment of the recovery trajectory, offering insights that static measurements alone cannot provide in assessing the speed and quality of visual rehabilitation [56]. Even though all comorbidity groups ultimately achieve comparable visual benefit from cataract surgery, patients with ocular comorbidities had slower early recovery, potentially indicating a need for closer monitoring in the early postoperative period. Therefore, ocular comorbidities, despite their small prevalence in the sample, can be interpreted as an important influencing factor. It is important, however, to note that our findings should be seen as a directional signal rather than a precise effect-size estimate while also identifying ocular comorbidity as a principal cause for delayed early visual recovery in our cohort [39].

Although all comorbidity groups ultimately attain broadly comparable final visual benefit from cataract surgery, the dynamic-recovery metric was selected so that any differential signal between comorbidity groups could be captured at the most informative point of the recovery curve. When interpreted at this dynamic level, ocular comorbidity emerged as the principal directional contributor to early-recovery differences across comorbidity groups. The pattern observed in our cohort is best read as a directional signal warranting closer early postoperative monitoring in patients with ocular comorbidities, rather than as a precise magnitude estimate, given the relatively limited size of the ocular-only subgroup [39]. This positioning is consistent with the multinational register-level evidence on cataract outcomes [4,27] and with the more focused evidence on ocular comorbidity populations such as glaucoma patients [57] and uniquely integrates both.

In contrast, systemic comorbidities alone did not appear to have a significant impact on early visual outcomes in our study. This corresponds with earlier clinical studies that showed no impact of those comorbidities on postoperative visual acuity [36] but is in contrast to some studies that connected diabetes and cardiovascular comorbidities with worse postoperative visual acuity [55,58]. Regarding specific systemic comorbidities that have become increasingly prevalent, such as autoimmune diseases and thyroid disorders, the literature suggests that autoimmune disease had no impact on postoperative visual results [29]. For thyroid disease, there is some possible connection with worse postoperative visual outcomes [59]. The problem with studies analysing postoperative visual acuity is that they did not specifically mention whether the patients had a certain systemic comorbidity [56].

In our analysis, we wanted to identify potential predictors of visual acuity outcomes that can inform future clinical practice regarding patient risk-stratification and a tailored follow-up approach. Factors included were a combination of common demographic (age, sex) and clinical parameters (comorbidity burden defined by patient’s group, endothelial cell count). Using ANCOVA, with age, sex, and endothelial cell count as covariates, we found no significant association of these covariates with either first (VA1) or final (VA2) postoperative visual acuity. Patient group was not significantly associated with VA1, but became a significant predictor of VA2, suggesting that comorbidity burden may become a more relevant factor for visual outcomes over a longer follow-up period. The lack of association for age and endothelial cell count appears contradictory to what has already been explored regarding the influence of age [60,61] and endothelial cell count [43]. Our study has the strength of involving a full cohort and not a selection of patients from everyday practice, thus mimicking the real-world context as closely as possible and avoiding over-emphasising the influence of a single factor such as age or endothelial cell count.

Our study has several limitations that should be considered when interpreting the results. An important limitation is the level of heterogeneity present in our study regarding the comorbidity status of included patients, where patients suffered from both ocular and systemic comorbidities and a combination of them. Furthermore, although the timeframe within which patients underwent surgery was very narrow, heterogeneity was introduced by including multiple surgeons who use different techniques, as well as ophthalmologists with varying levels of experience and methodology for postoperative evaluations—including the qualitative, slit-lamp-based categorisation of postoperative corneal status as transparent, less-transparent, or with Descemet folds, recorded by 10 different operating ophthalmologists, which follows the categorical scheme used by Aynala and Pradeep in their 2024 comparative outcome study of immediate postoperative corneal oedema in a teaching-hospital setting [36]. Pachymetric and OCT-based measurements remain the research-grade gold standard [32] but were not deployable at the scale required for this retrospective design given the patient volume and the limited capacity available for protocol-driven imaging in our regional service. The retrospective design also led to heterogeneity in follow-up schedules, where patients were followed up after 8–10 days of surgery for the first visit, while the number of days for the final visit varied even more. Early postoperative acuity was operationally defined as the first Snellen measurement the patient was able to produce rather than at a pre-specified calendar day, so that early postoperative corneal oedema could in principle shift the documented VA1 by a few days relative to a fixed day-8 schedule. The magnitude of this displacement is bounded because clinical corneal status did not differ significantly between comorbidity groups, so chart-readability-related VA1 delay is approximately equally distributed across the four comparison arms. Furthermore, if a more-oedematous patient is given more calendar time before the first measurable Snellen reading, they would in principle have had more time to recover, yet the only-ocular-comorbidities group (which carries the highest burden of corneal involvement in routine practice) still showed the worst early-recovery trajectory in our data, so any residual timing bias acts conservatively and cannot exaggerate the reported early-recovery deficit. The University Hospital of Split is the only tertiary ophthalmology referral centre for approximately one million Croatian residents and approximately 500,000 inhabitants of the southern part of neighbouring Bosnia and Herzegovina, with a sizeable transient tourist population during the summer season, so the variability in follow-up dates reflects the routine clinical workflow of a public-health regional service in which postoperative visits are scheduled on individual clinical grounds rather than at fixed calendar intervals.

All these aspects are very important to have in mind when exploring the relationship of postoperative corneal status and visual acuity results with relevant risk factors, as they have already been shown to be statistically relevant individually. Finally, it is important to note that a certain proportion of patients had missing visual acuity levels and were nevertheless included in the analysis for other factors in order to maintain the integrity of our dataset.

However, we believe that this approach can, at the same time, be advantageous since it provides a certain degree of sensitivity analysis to the extent that comorbidities burden influences the outcomes in an uncensored context. By including a diverse patient population and multiple practitioners, the study achieves high ecological validity, reflecting routine clinical practice more accurately than highly controlled trials with selected patients and single surgeons [4,55].

Using data from all available patients, this study effectively captures the real-world setting of a public health system where patients with various combinations of both ocular and systemic comorbidities are assigned the same treatment approach. While knowledge about interactions between individual comorbidities and surgery outcomes is very relevant and important, our analysis shows that evaluating the interplay of multiple conditions rather than isolated comorbidities is more applicable to everyday ophthalmic care and helps avoid over-emphasising the weight of any single risk factor [12,23]. However, we believe that this approach can, at the same time, be advantageous since it provides a certain degree of sensitivity analysis to the extent that comorbidities burden influences the outcomes in an uncensored context. By including a diverse patient population and multiple practitioners, the study achieves high ecological validity, reflecting routine clinical practice more accurately than highly controlled trials with selected patients and single surgeons [4,55].

## 5. Conclusions

In conclusion, cataract surgery results in significant visual improvement regardless of the presence of systemic and/or ocular comorbidities. While ocular comorbidities may have been associated with delayed early postoperative recovery, long-term visual outcomes did not seem to depend on the coexistence of systemic and/or ocular comorbidities. These findings are consistent with the existing literature and support the need for a tailored postoperative approach that focuses follow-up on high-risk patients while allowing patients with a low risk of poor outcome a more lenient follow-up approach. These measures may help reduce unnecessary follow-up burden, ultimately optimising resource utilisation by directing resources towards patients who need it the most [8,9,10,62]. Finally, this real-world data study can serve as a foundation for future studies aimed at developing standardised protocols for the individualised preoperative assessment and postoperative management of patients with complex medical backgrounds. Establishing such protocols may lead to improved outcomes while enhancing the efficiency and cost-effectiveness of ophthalmic care delivery.

## Figures and Tables

**Table 1:** Clinical characteristics of patients undergoing cataract surgery.

Parameter	No Ocular and/or Systemic Comorbidities (n)	With Systemic and Ocular Comorbidities (n)	With Systemic/Without Ocular Comorbidities (n)	Without Systemic/with Ocular Comorbidities (n)	*p* Value
Age, median (IQR)	n = 61 67.0 (63.8–74.3)	n = 59 76.0 (71.3–81.0)	n = 182 74.0 (69.0–79.0)	n = 13 70.0 (63.3–81.0)	<0.001
Gender, No (%)					
Male, No. (n = 125, 39.7%)	29	19	68	9	0.044
Female, No. (n = 190, 60.3%)	32	40	114	4	
Endothelial cell count (cells/mm^2^), median (IQR)	n = 56 2374.00 (2175.00–2617.00)	n = 57 2302.00 (1817.25–2468.00)	n = 172 2419.50 (2113.50–2629.50)	n = 12 2351.00 (2017.00–2469.50)	0.130

Values for age and endothelial cell count are shown as median (interquartile range). Overall, 18 patients had missing endothelial cell count and as such were excluded from corresponding analysis. Statistical analysis was performed using the Kruskal–Wallis test. Values for gender are shown as counts and percentages, so the statistical analysis was performed using the chi-square (χ^2^) test.

**Table 2:** Association of the corneal status in patients undergoing cataract surgery.

Parameter	No Ocular or Systemic Comorbidities (No.)	With Systemic and Ocular Comorbidities (No.)	With Systemic but Without Ocular Comorbidities (No.)	With Ocular but Without Systemic Comorbidities (No.)	*p*-Value
**Overall corneal status**					
Pathological cornea	12	22	37	4	0.062
Healthy cornea	48	37	139	9	
**Corneal clinical status**					
Transparent cornea	48	37	139	9	0.218
Non-transparent cornea	3	4	5	1	
Descemet folds	9	18	32	3	

Values are shown as counts and compared using the chi-square (χ^2^) test. The bold values in the table represent two different methods of grading postoperative corneal oedema.

**Table 3:** Visual acuity outcomes (median, interquartile range) in groups of patients who underwent cataract surgery.

Group (n)	VA1 †	VA2 ‡
No ocular or systemic comorbidities	n = 51, 0.137 (0.06–0.20)	n = 44, 0.04 (0.00–0.11)
With systemic and ocular comorbidities	n = 54, 0.27 (0.16–0.4)	n = 52, 0.13 (0.04–0.30)
With systemic but without ocular comorbidities	n = 154, 0.16 (0.1–0.3)	n = 149, 0.04 (0.00–0.16)
With ocular but without systemic comorbidities	n = 11, 0.30 (0.17–0.46)	n = 10, 0.13 (0.00–0.30)

Abbreviations: VA1 (visual acuity on the first postoperative visit), VA2 (visual acuity on the last postoperative visit). † Kruskal–Wallis test, *p* = 0.0002, the group that has no comorbidities has statistically lower VA1 levels than the group that has both comorbidities, as well as the group that has only ocular comorbidities. Also, a statistically higher value was observed in the group with both systemic and ocular comorbidities compared to the group with systemic but no ocular comorbidities (Post hoc Dunn test). ‡ Kruskal–Wallis test, *p* = 0.0001, the group that has no comorbidities has statistically lower VA2 levels than the group that has both systemic and ocular comorbidities. The group that had only systemic comorbidities had significantly lower VA2 levels than the group that had both comorbidities (Post hoc Dunn test).

**Table 4:** Visual acuity recovery (median, interquartile range) in patients who underwent cataract surgery.

Group (n)	VA1–VA0 †	VA2–VA0 ‡
No ocular or systemic comorbidities	n = 50 −0.329 (−0.542 to −0.241)	n = 43 −0.398 (−0.674 to −0.345)
With systemic and ocular comorbidities	n = 54 −0.320 (−0.796 to −0.154)	n = 52 −0.389 (−0.729 to −0.301)
With systemic but without ocular comorbidities	n = 151 −0.320 (−0.495 to −0.194)	n = 146 −0.398 (−0.602 to −0.301)
With ocular but without systemic comorbidities	n = 11 −0.176 (−0.294 to −0.049)	n = 10 −0.311 (−0.477 to −0.137)

Abbreviations: VA1–VA0 (visual acuity recovery between first postoperative visit and preoperative values), VA2–VA0 (visual acuity recovery between last postoperative visit and preoperative values). † Kruskal–Wallis test, *p* = 0.031. Patients without systemic but with ocular disease were statistically different from the other three groups (Post hoc Dunn test). ‡ Kruskal–Wallis test, *p* = 0.242.

## Data Availability

The data used in this study are derived from patient medical records and contain potentially identifiable clinical information. Therefore, due to ethical, legal, and institutional restrictions regarding patient confidentiality, the dataset cannot be made publicly available. However, de-identified data can be made available from the corresponding author upon reasonable request, subject to approval by the relevant institutional and ethical regulations.

## Abbreviations

The following abbreviations are used in this manuscript:

PHACO	Phacoemulsification
PEX	Pseudoexfoliation syndrome
VA0	Preoperative visual acuity
VA1	First Postoperative visual acuity
VA2	Final Visual Acuity
VA1–VA0	Change from preoperative visual acuity to first postoperative visual acuity
VA2–VA0	Change from preoperative visual acuity to final postoperative visual acuity
